# Biomarkers of Induced Active and Passive Smoking Damage

**DOI:** 10.3390/ijerph6030874

**Published:** 2009-02-26

**Authors:** Maura Lodovici, Elisabetta Bigagli

**Affiliations:** Department of Pharmacology, University of Florence, Viale Pieraccini 6, 50139, Florence, Italy

**Keywords:** Tobacco smoking, biomarkers, carcinogenic DNA adducts, genetic polymorphisms, cancer risk

## Abstract

In addition to the well-known link between smoking and lung cancer, large epidemiological studies have shown a relationship between smoking and cancers of the nose, oral cavity, oropharynx, larynx, esophagus, pancreas, bladder, kidney, stomach, liver, colon and cervix, as well as myeloid leukemia. Epidemiological evidence has reported a direct link between exposure of non-smokers to environmental tobacco smoke and disease, most notably, lung cancer. Much evidence demonstrates that carcinogenic-DNA adducts are useful markers of tobacco smoke exposure, providing an integrated measurement of carcinogen intake, metabolic activation, and delivery to the DNA in target tissues. Monitoring accessible surrogate tissues, such as white blood cells or bronchoalveolar lavage (BAL) cells, also provides a means of investigating passive and active tobacco exposure in healthy individuals and cancer patients. Levels of DNA adducts measured in many tissues of smokers are significantly higher than in non-smokers. While some studies have demonstrated an association between carcinogenic DNA adducts and cancer in current smokers, no association has been observed in ex or never smokers. The role of genetic susceptibility in the development of smoking related-cancer is essential. In order to establish whether smoking-related DNA adducts are biomarkers of tobacco smoke exposure and/or its carcinogenic activity we summarized all data that associated tobacco smoke exposure and smoking-related DNA adducts both in controls and/or in cancer cases and studies where the effect of genetic polymorphisms involved in the activation and deactivation of carcinogens were also evaluated. In the future we hope we will be able to screen for lung cancer susceptibility by using specific biomarkers and that subjects of compared groups can be stratified for multiple potential modulators of biomarkers, taking into account various confounding factors.

## 1. Introduction

Tobacco is the single most preventable cause of death in the world today, it has been estimated to have killed more than five million people in 2008 and will be responsible for the death of more than eight million by 2030 [1]. While many countries have adopted consistent policies against its use in public places, there are still approximately 1.3 billion smokers in the world and hundreds of millions of smokeless tobacco users. Cigarette smoking causes 30% of all cancer mortality in developed countries, and smokeless tobacco use is an important cause of cancer, particularly in southern Asia [2]. Tobacco smoke contains more than 4,000 chemicals and some of them are carcinogens. The strongest carcinogens present in tobacco smoke are polycyclic aromatic hydrocarbons (PAH), *N*-nitrosamines, aromatic amines, aldehydes, benzene and butadiene. Cigarette smoke products can be divided into particulate and gas phases. The particulate phase contains nicotine, nitrosamines [4-(methylnitrosamino)-1-(3-pyridyl)-1-butanone], *N*-nitrosonornicotine, metals, polycyclic aromatic hydrocarbons (PAH) and carcinogenic amines (4-aminobiphenyl). The gas phase contains carbon monoxide, carbon dioxide, benzene, ammonia, formaldehyde, hydrogen cyanide, *N*-nitroso-dimethylamine and *N*-nitrosodiethylamine [3].

In addition to the well-known link between smoking and lung cancer, large epidemiological studies have shown a relationship between smoking and cancers of the nose, oral cavity, oropharynx, hypopharynx, larynx, esophagus, pancreas, bladder, kidney, stomach, liver, colon, cervix as well as myeloid leukemia [4]. Cigarette smoking predisposes the individual to several different clinical atherosclerotic syndromes, including stable angina, acute coronary syndromes, aortic and peripheral atherosclerosis sudden death, and stroke [5].

## 2. Environmental Tobacco Smoke

In 1986 the Surgeon General of the United States published a landmark report, based on epidemiological evidence, asserting a direct link between exposure of non-smokers to environmental tobacco smoke and disease, most notably, lung cancer [6]. In the same year, the National Academy of Sciences reported similar conclusion regarding the adverse effects of exposure to environmental tobacco smoke [7]. In 1997, the California Environmental Protection Agency published the final draft of a report regarding all known health effects of exposure to environmental tobacco smoke, including ischemic heart disease, lung cancer and bronchitis [8]. More than 50 studies on passive smoking and lung cancer risk in never smokers, especially spouses of smokers, have been carried out and published within the past 25 years. These studies show that there is a statistically significant and consistent association between lung cancer risk in spouses of smokers and second-hand smoke from the spouse who smokes. This excess risk is on the order of 20% for women and 30% for men. The excess risk increases with increasing exposure [9]. It has been described that there is an increased risk of lower respiratory diseases in children of smoking parents and an increased risk of asthma [8]. The results from these reports have increased the debate on smoking and environmental tobacco smoke’s health impact on non-smokers and set off controversy regarding smoking in the workplace and public buildings. Tobacco combustion results in the formation of mainstream smoke and sidestream smoke. Cigarette smoke that is drawn through the tobacco into an active smoker’s mouth is known as mainstream smoke. Sidestream cigarette smoke is the smoke emitted from the burning end of a cigarette.

Environmental tobacco smoke results from the combination of sidestream smoke (85%) and a small fraction of exhaled mainstream smoke (15%) from smokers. For the most part, the chemical compositions of sidestream smoke and mainstream smoke are qualitatively similar and most toxic carcinogens are present in both of them but in different concentrations because of ageing and dilution with ambient air. Smokers who actively inhale very large doses of mainstream smoke-carcinogens, have a higher intake of carcinogens than environmental tobacco smoke-exposed individuals [10,11].

## 3. Environmental Exposure, Internal Dose and Biologically Effective Dose

Molecular biomarkers are typically indicators of exposure effect or susceptibility [12]. A biomarker of exposure indicates the presence of exposure to an environmental agent while a biomarker of effect indicates the presence of a biological response to exposure to an environmental agent. Biomarkers thus have significant potential in clarifying the relationship between environmental agents and disease [13]. A biomarker of exposure requires measurement of toxicant levels in the environment and characterization of the individual presence, and interaction with that environment. But, a complexity arising from the use of ambient measurements to determine exposure status of individuals is the heterogeneous nature of most environmental contaminations. These measurements should be added to a system that integrates fluctuating exposures over time and relates time of exposure to dose. Given these problems of extrapolating ambient measurements to specific individual exposure, it has been well recognized that measures of the internal dose of a specific agent provide a clearer demonstration that a toxicant has been absorbed and possibly distributed in the body. Internal dose measurements are an effective identification of previous exposure, however, they do not provide evidence that toxicological damage has occurred. Among the various possible biomarkers of cancer risk is the measurement of carcinogen-DNA adducts, which are direct products of, or surrogate markers for, damage to critical macromolecular targets. DNA adduct measurement provides an integrated measurement of carcinogen intake, metabolic activation and delivery to the target macromolecule in target tissues (biologically effective dose) [14].

Many different type of analytical techniques have been developed to measure DNA adducts, including ^32^P-postlabeling with or without nuclease P1, immunoassay using antibodies to DNA adducts, physicochemical properties of adducts, such as fluorescence and mass spectrometry. Although each methodology has very different detection endpoints, the results obtained show a high quantitative similarity [15–17].

## 4. Biomarkers of Active and Passive Smoke Exposure

Since the aim of this review is the evaluation of carcinogenic-DNA adducts as biomarkers of tobacco smoke exposure and its carcinogenic activity we reported studies that measured smoking-related DNA adducts as biomarkers of tobacco smoke exposure both in cancer cases and healthy smokers and non-smoker subjects [18–25]. The results obtained from these studies demonstrate that, as reported in Table 1A, higher DNA adduct levels were found for tobacco smoke exposure (lung, bronchus, uterine cervix. *etc*.) in target organs from smokers than non smokers. Some of these authors also found a correlation between adduct levels and smoke exposure [18,20]. In one study a good correlation was also found between DNA adducts and benzo(a)pyrene levels measured in lung tissue from smokers [24].

When a surrogate tissue (white blood cells) was used, different results were observed from various laboratories. Some studies indicated a correlation between adduct levels in blood cells and lung as well as higher DNA adducts in smokers than in non smokers [26–28], while others demonstrate that white blood cells are not a good surrogate tissue [29,30] and still others that similar DNA adduct levels occur in blood cells from smokers and non-smokers [31,32], using 8-hydroxydeoxyguanosine as a marker of oxidative DNA damage [25,33]. With the same method other authors [34,35] report a significant increase in oxidative DNA damage in leukocytes from subjects exposed to environmental tobacco smoke and a correlation between DNA damage and tobacco exposure, measured by plasma cotinine levels. Using DNA isolated from bronchoalveolar lavage (BAL) cells, DNA adduct levels were found to be significantly higher in smokers than in non-smokers [36–39]. But in 1992 Alexandrov *et al.* [20] published an important observation demonstrating that benzo[a]pyrenediol epoxide-DNA adducts were positively correlated with CYP1A1 enzyme activity in lung tissue of smokers. Later, many researchers focused their attention on the effect of genetic polymorphisms involved in the activation and deactivation of carcinogens on risk damage to tobacco exposure.

In our review we also considered studies that took into account the effect of genetic polymorphisms involved in the activation and deactivation of carcinogens on DNA adduct levels. For this reason, since major classes of carcinogens present in tobacco smoke are converted into DNA reactive metabolites by cytochrome P450-related enzymes, we reported studies that evaluated the effect of polymorphisms of CYPs in humans, alone or in addition to phase II enzymes, particularly glutathione S-transferase (GST) on DNA adduct levels. These data are summarized in Table 1B. CYP1A1 is a phase I enzyme involved in the metabolic activation of aromatic amines and PAH and may affect the metabolism of environmental carcinogens and alter susceptibility to lung cancer. On the contrary, GST is a large family of phase II enzyme involved in xenobiotic detoxification. Butkiewicz *et al.* [40] found higher adduct levels in lung tissue from smokers lung cancer than non-smokers and a significant relationship between high adduct levels and the combined GSTM1 (null) and CYP1A1 polymorphic genotype. Similarly, Lewis *et al.* [41] reported that adduct levels tended to be higher in individuals with GSTM1 *null*, GSTT1 *null* or GSTP1 *wt* genotypes in bronchial lavage samples while Peluso *et al.* [42] found no significant effect on DNA adducts and genetic polymorphisms of CYP1A1, GSTM1 and GSTT1 in 55 nasal brushing and bronchoscopies. Texeira *et al.* [43] reported that lymphocytes from smokers had significantly higher DNA adducts than non-smokers with a good correlation between the levels of DNA adducts and the number of cigarettes smoked. The levels of DNA adducts in smokers is dependent on polymorphisms of CYP1A1 MspI. In fact, the allele variant of CYP1A1 MspI had DNA adducts about two-fold higher than CYP1A1 MpsI with no allele variant, but no effect was observed for the GST genotypes studied [43]. On the contrary, DNA adduct levels, adjusted for the number of cigarettes smoked per day, were found to be significantly higher in mononuclear blood cells from individuals with GSTM1 *null* than those with GSTM1 active [44]. In one study where smokers were divided into a high-risk group with CYP1A1 MspI and/or exon 7 Ile462Val allele variants, glutathione S-transferase M1 (GSTM1*) null* allele and *wt* glutathione S-transferase P1 (GSTP1), and a low-risk group (*wt* CYP1A1) and higher deactivation capacity (active GSTM1, GSTP1 Ile105Val allele), significantly lower BPDE-DNA adduct levels were reported in low-risk group [45]. Interestingly, Van Schooten *et al*., [46] found that MPO mutant genotypes are associated with reduced MPO activity in BAL fluid and reduced smoking-related DNA adduct levels in BAL cells in a gene-dose manner. Ketelslegers *et al.* [47] observed that GSTM1*0, mEH*2, and GPX1*1 are the most relevant polymorphisms for lymphocytic DNA adduct levels in smokers. Individuals having four risk alleles for these three genes had higher DNA adduct levels than individuals not possessing these particular risk alleles. However, recently Mollerup *et al.* [48] found lung DNA adducts highly related to CYP1A1 expression, but irrespective of smoking-status in cancer cases.

## 5. Biomarkers of Tobacco Smoke Carcinogenic Activity

In the molecular epidemiological literature carcinogen-DNA adducts are referred to as biomarkers of the biologically effective dose of a carcinogen and thus biomarkers of cancer risk. Carcinogen-DNA adducts represent the amount of carcinogen absorbed by the body that is not detoxificated, that is bound to cellular macromolecules and has not been repaired [49,50]. There is clear evidence that carcinogen-DNA adducts can reflect exposure to xenobiotics and there is clear mechanistic evidence that carcinogen-DNA adduct formation is a key to chemical carcinogenesis [51–53]. Recently, a number of reviews on carcinogen-DNA adducts mention epidemiological studies that have investigated whether increased adduct levels are associated with cancer incidence [54–56]. A minority of these reports takes into account the limitations of this literature from an epidemiological point of view, eg. short half-lives of adducts, multiple exposures, weak effects and interactions with genetic susceptibility [53,55]. Veglia *et al*. [55] conducted a meta-analysis of active smoking and cancer, including five studies of lung cancer [57–60,27], one of oral cancer [61] and one of bladder cancer [62] in which bulky DNA adducts were measured. They found a significant relationship between bulky DNA adducts and cancer in current smokers, while no association was observed in ex- and never smokers. The methods and results of the individual studies analyzed in the meta-analysis of Veglia *et al.* [55] are reported in Table 2A.

Hou *et al.* [58] found no association between carcinogen-DNA adducts and cancer in smokers but the only matching factor accounted for in these analyses was smoking. However, it was previously reported that failure to fully account for matching in a case-control study tends to bias results toward the null [63]. The same bias was also present in the study of Vulimiri *et al.* [59] that found significantly higher carcinogen-DNA adducts in cases than in controls, although no statistical control for the matching variables was considered and the observed difference may be an underestimate of the effective differences. In addition to the studies included in the meta-analysis by Veglia *et al.* [55] data obtained from three other studies have been published on smoking-related cancers and carcinogen-DNA adducts (Table 2A).

Adduct levels in bladder tissue were measured by ^32^P-postlabeling by Benhamou *et al.*, [64] and no significant association was found between adducts and bladder cancer case-control status. However, smoking status was not taken into account in the data analysis. In the study of Peluso *et al.* [65] a relationship was observed between detectable adducts and lung cancer risk among never-smokers with OR = 4.04 (1.06–15.42), while adduct levels were not significantly associated with lung cancer in former-smokers. For the other cancers no association with detectable adduct levels was found [64]. On the contrary, Bak *et al.* [66] measured adduct levels in white blood cells from 255 randomly selected subjects considering sex, year of birth and smoking duration and demonstrated that among current smokers high adducts were significantly associated with lung cancer risk (incidence rate ratio (IRR) = 1.61 (1.04–2.49) while high adduct levels, defined as being above the median level of the controls, were not.

Although tobacco smoking is a well established risk factor for tobacco-related cancers, not all of those who have been exposed will develop disease, suggesting that there is individual variation in cancer susceptibility in the general population and that genetic polymorphisms may modulate the association observed between exposure and cancer [67,3] Some of the most widely studied polymorphic loci are those coding for phase I and II enzymes involved in the activation and conjugation of carcinogens from tobacco smoke. The most frequently studied include CYP1A1, microsomal epoxide hydrolase 1 (mEH/EPHX1), myeloperoxidase (MPO), NAD (P) H quinone oxidoreductase 1 (NQO1) and the glutathione S-transferases (M1, P1 and T1), although others have also been studied [3]. Individuals with some genetic variants in the GST and CYP genes are reported to have different levels of PAH-DNA adducts in their lung tissue than those with *wt* genotype, and genetic variants have been extensively studied as candidates for lung cancer susceptibility as summarized in Table 2B. Ryberg *et al.* [68], analyzing 70 lung cancers, found higher DNA adduct levels in patients with the null GSTM1 genotype than in those with at least one intact GSTM1 allele. Moreover, significantly lower adduct levels were found in patients with the polymorphic GSTP1 genotype (with higher affinity for PAH-diolepoxides) compared to those with the wild type GSTP1 genotype. The same results were obtained by Royas *et al.* [69] who found a highly significant difference in DNA adduct levels between lung cancer patients with GSTM1 *null* and those with GSTM1 active genotype. On the contrary, Schoket *et al.* [70] reported no association between the combined CYP1A1 MspI and GSTM1 genotypes and DNA adduct levels in bronchial tissue from 150 pulmonary surgery patients (126 with lung cancer). In one study analyzing 73 cancer cases Cheng *et al*. [60] found significantly high adducts levels than controls (33), but no association was observed with CYP1A1Msp1 or GSTM1 genotypes; on the contrary data reported by Li *et al*. [71] demonstrated an association between high adducts and CYP1A1 polymorphism in pancreatic cancer patients.

## 6. Conclusions

The available data obtained from past decades show that DNA adduct formation is a key step in tobacco carcinogenesis, although the experimental evidence that carcinogen-DNA adducts are useful as biomarkers for evaluating the association between tobacco exposure and cancer risk is not always recognized. Hopefully in the future we will be able to screen individuals for cancer susceptibility by using specific biomarkers (for precise DNA adducts and metabolic polymorphisms) and then subjects can be stratified for multiple potential modulators of biomarkers, taking into account various confounding factors.

## Figures and Tables

**Table 1A.** Relationship between smoking and carcinogenic-DNA adducts in target organs due to tobacco smoke.

Source of DNA	Methods	Higher DNA adducts in smokers than ex-and non-smokers
Lung [18]	^32^P-postlabeling	29 cancer cases (17 smokers, 7 ex-smokers, 5 non-smokers)
Bladder [19]	^32^P-postlabeling	39 healthy subjects (18 smokers, 21 non-smokers)
Lung [20]	HPLC/fluorescence, ^32^P-postlabeling	13 cancer cases (11 smokers, 2 ex-smokers)
Uterine cervix [21]	^32^P-postlabeling	16 HPV (10 smokers, 6 non-smoker)
Lung [22]	^32^P-postlabeling, fluorescence	39 cancer cases (26 smokers, 11 ex-smokers, 2 non-smokers)
Pancreas [23]	^32^P-postlabeling	20 cancer cases (10 smokers, 10 non-smokers) 24 controls
Lung [24]	HPLC/fluorescence	39 (12 smokers, 6 ex-smokers, 21 non-smokers)
Lung [25]	HPLC/ECD detection of 8-oxo-dG	30 healthy subjects (14 smokers, 7 ex-smokers, 9 non-smokers)

**Table 1B.** Relationship between smoking and carcinogenic-DNA adducts in cancer-target organs due to tobacco smoke and influence of metabolic genotypes.

Source of DNA	Methods	Elevated DNA adducts in smokers than non-smokers
Lung [40]	^32^P-postlabeling	High adduct levels associated with CYP1A1 among subjects with GSTM1 *null*
Lung [41]	^32^P-postlabeling	High adducts in individuals with GSTM1 *null*, GSTT1 *null* or GSTP1 wt
Lung [42]	^32^P-postlabeling	In smokers increased adduct levels in both nasal mucosa and lymphocytes. No significant effect of CYP1A1, GSTM1 and GSTT1
Lymphocytes [43]	^32^P-postlabeling	In smokers with CYP1A1 MspI allele variant. Adduct levels no influenced by GST genotypes

Mononuclear cells [44]	^32^P-postlabeling	In smokers with GSTM1 *null* (adjusted for the amount of cigarettes smoked per day) and in slow acetylators for both NAT1 and NAT2 with GSTM1 *null* than fast acetylator with GSTM1 (+)

Leukocytes [45]	HPLC/fluorescence	In smokers with high risk genotype (CYP1A1 allele variant, GSTM1 *null*, and GSTP1 wt)
BAL fluid and cells [46]	^32^P-postlabeling	In smokers with MPO wt than MPO mutant genotype. The effect is gene dose-dependent

Lymphocytes [47]	^32^P-postlabeling	In smokers with GSTM1 *null*, mEH*2 and GPX1*1
Lung [48]	^32^P-postlabeling	In individuals with high CYP1A1 expression, but irrespective of the smoking status

**Table 2A.** Relationship between smoking-related DNA adducts in tissues of cancer cases and/or controls.

Cancer	Methods	Relationship
Lung [57]	PAH-DNA adducts (white blood cells) ELISA	Strong OR= 7.7 (1.7–34)
Lung [58]	Bulky DNA adducts (lymphocytes) ^32^P-postlabeling	None
Lung [59]	Bulky DNA adducts (lymphocytes) ^32^P-postlabeling	Present
Lung [60]	Bulky DNA adducts (lung tissue) ^32^P-postlabeling	Strong OR= 25.19 (2.99–211.99)
Lung [27]	Bulky DNA adducts (white blood cells) ^32^P-postlabeling	OR= 2.98 (1.05–8.42)
Oral [61]	Bulky DNA adducts (white blood cells) ^32^P-postlabeling	None
Bladder [62]	Bulky DNA adducts ((white blood cells) ^32^P-postlabeling	Strong OR= 5.25 (2.21–12.43)
Bladder [64]	Bulky DNA adducts (bladder tissue) ^32^P-postlabeling	None
Lung, upper respiratory, bladder and leukemia [65]	Bulky DNA adducts (white blood cells) ^32^P-postlabeling	None
Lung [66]	Bulky DNA adducts (white blood cells) ^32^P-postlabeling	Present

**Table 2B.** Relationship between smoking-related DNA adducts in tissues of cancer cases and/or controls and effect of metabolic polymorphisms.

Tissue	Methods	Number of subjects	Elevated DNA adducts and metabolic polymorphism influence
Lung [68]	^32^P-postlabeling	70 cancer cases (smokers)	In patients with GSTM1 *null* or GSTP1 wt than those with GSTM1 *positive* or GSTP1 polymorphic (n=70)
Lung [69]	HPLC/fluorescence	20 cancer cases (smokers)	In patients (n=20) with GSTM1 *null* than those with GSTM1 (+)
Bronchus [70]	^32^P-postlabeling	124 cancer cases (70 smokers, 40 ex-smokers non-smokers 14) 26 controls (12 smokers, 5 ex-smokers, 9 non-smokers)	In cancer case than in controls. Adducts levels were not influenced by CYP1A1 or GSTM1 polymorphism
Lung [60]	^32^P-postlabeling	73 cancer cases (32 smokers, 38 non-smokers) 33 controls (11 smokers, 22 smokers)	In cancer cases than in controls, but not higher in smokers than non-smokers. Adducts not influenced by CYP1A1Msp1 or GSTM1 genotypes
Pancreas [71]	^32^P-postlabeling and HPLC/ECD detection of 8-oxo-dG	31 cancer cases 11 controls	In cancer cases than in controls. Association of DNA adducts with CYP1A1 polymorphism
